# The Major Amputations in the Cook County Hospital during the Eleven Years Ending December, 1875

**Published:** 1877-10

**Authors:** C. T. Fenn

**Affiliations:** Chicago


### THE MAJOR AMPUTATIONS IN THE COOK COUNTY HOSPITAL DURING THE ELEVEN YEARS ENDING DECEMBER, 1875.

By C. T. Fenn, M. D., Chicago.

In the latter part of December, 1876, the old County Hospital, occupied since January, 1866, was vacated, and the patients were removed to the new hospital. This departure from filth and miasma to cleanliness and wholesome surroundings, inaugurates a new period in the history of the County Hospital; and all statistical reports, which in the future may be collected from the records of the hospital, must always bear in mind the difference of the sanitary conditions during the first and second period. In view of these facts, we prepared the following list of all the major amputations performed in the old hospital from 1866 to 1876.

The table does not include amputations of the hand and foot, or disarticulations of the wrist and the ankle joints.

*Primary amputations* are those which were done within the first forty-eight hours succeeding the injury.

*Intermediate amputations* are those which were performed between the third and thirtieth days.

*Secondary amputations* are those which were done after the thirtieth day.

*Pathological amputations* are those which were undertaken for the cure of disease.

### CAUSES LEADING TO AMPUTATION.

There were fourteen amputations for ill-conditioned stumps; one in the upper extremity; thirteen in the lower extremity. All were, more or less, remote from the seat of original operation: one being at the hip-joint for malignant disease of leg-stump; one in the middle third of the thigh for secondary

TABLE OF MAJOR AMPUTATIONS IN THE COOK COUNTY HOSPITAL, FROM 1866 TO 1876.

| | PRIMARY. | | | INTERMEDIATE. | | | SECONDARY. | | | PATHOLOGICAL. | | | NOT CLASSIFIED. | | | TOTAL. | | |
|---|---|---|---|---|---|---|---|---|---|---|---|---|---|---|---|---|---|---|
| | Cases. | Deaths. | Per Cent. | Cases. | Deaths. | Per Cent. | Cases. | Deaths. | Per Cent. | Cases. | Deaths. | Per Cent. | Cases. | Deaths. | Per Cent. | Cases. | Deaths. | Per Cent. |
| ARM. | | | | | | | | | | | | | | | | | | |
| Shoulder joint | 7 | 4 | | | | | | | | | | | | | | 7 | 4 | |
| Upper third | 9 | 2 | | 1 | | | | | | 2 | | | | | | 12 | 2 | |
| Middle third | 1 | | | | | | 1 | | | 1 | | | | | | 3 | | |
| Lower third | 5 | | | 3 | | | 1 | | | 3 | 1 | | | | | 12 | 1 | |
| Seat not recorded | 4 | | | | | | | | | | | | 1 | | | 5 | | |
| In all | 26 | 6 | 23.07 | 4 | | | 2 | | | 6 | 1 | | 1 | | | 39 | 7 | 17.94 |
| FOREARM. | | | | | | | | | | | | | | | | | | |
| Elbow joint | 1 | | | | | | | | | | | | | | | 1 | | |
| Upper third | 4 | 2 | | | | | | | | | | | | | | 4 | 2 | |
| Middle third | 1 | | | | | | | | | 1 | | | | | | 2 | | |
| Lower third | 3 | | | 2 | 1 | | 1 | | | | | | | | | 6 | 1 | |
| Seat not recorded | 1 | | | | | | | | | | | | | | | 1 | | |
| In all | 10 | 2 | 20.00 | 2 | 1 | | 1 | | | 1 | | | | | | 14 | 3 | 21.42 |
| THIGH. | | | | | | | | | | | | | | | | | | |
| Hip joint | | | | | | | | | | 3 | | | | | | 3 | | |
| Upper third | | | | | | | 1 | | | | | | | | | 1 | | |
| Middle third | 7 | 3 | | 1 | | | | | | 1 | | | | | | 9 | 3 | |
| Lower third | 11 | 6 | | 2 | | | | | | 14 | 3 | | | | | 27 | 9 | |
| Seat not recorded. | 1 | | | 1 | | | | | | 1 | | | 1 | | | 4 | | |
| In all | 19 | 9 | 47.36 | 4 | | | 1 | | | 19 | 3 | 15.78 | 1 | | | 44 | 12 | 27.27 |
| LEG. | | | | | | | | | | | | | | | | | | |
| Knee joint | 3 | 1 | | | | | 1 | | | 4 | | | | | | 8 | 1 | |
| Upper third | 13 | 4 | | 5 | 2 | | 8 | 2 | | 6 | 1 | | | | | 32 | 9 | |
| Middle Third | 4 | | | 4 | 1 | | | | | 2 | 1 | | | | | 10 | 2 | |
| Lower third | 3 | | | 9 | 4 | | 6 | 2 | | 9 | 1 | | | | | 27 | 7 | |
| Seat not recorded. | 5 | | | 2 | | | | | | 1 | | | 2 | | | 10 | | |
| In all | 28 | 5 | 17.85 | 20 | 7 | 35.00 | 15 | 4 | 26.66 | 22 | 3 | 13 63 | 2 | | | 87 | 19 | 21.83 |
| SUMMARY. | | | | | | | | | | | | | | | | | | |
| Upper extremity. | 36 | 8 | 22.22 | 6 | 1 | 16.66 | 3 | | | 7 | 1 | 14.28 | 1 | | | 53 | 10 | 18.86 |
| Lower extremity. | 47 | 14 | 29.78 | 24 | 7 | 29.16 | 18 | 5 | 27.77 | 39 | 5 | 12.82 | 3 | | | 131 | 31 | 23.66 |
| In all | 83 | 22 | 26.50 | 30 | 8 | 26.66 | 21 | 5 | 23.80 | 46 | 6 | 13.04 | 4 | | | 184 | 41 | 22.28 |

hemorrhage from leg-stump; six in the upper third of the leg, two of which were for ulcerated foot-stumps ; one for gangrene of foot-stump and leg ; and three for ulcerated leg-stump. One of the latter resulted in death, and one in a second re-amputation above the knee. Five in the lower third of the leg for unhealed leg-stumps, two, double, resulted in death; one in a second reamputation. One in the leg, seat not designated. One of the arm for inflammation of fore-arm-stump.

Mortality after re-amputations $21\frac{42}{100}$ per cent.

There were eight amputations for ulcers, all in the lower extremity.

There were eight amputations for malignant diseases. Three in the upper extremity ; five in the lower extremity.

There were ten amputations for frost-bite. One in the upper extremity, and nine in the lower extremity.

There were twenty-seven amputations for caries. Two in the upper extremity, and twenty-five in the lower extremity. Twenty-five of these cases involved the joints ; one the wrist, one the elbow, nine the ankle, twelve the knee, and two the hip. Of the nine cases of caries of the ankle-joint, one died ; of the twelve cases of caries of the knee, two died.

There were one hundred amputations for injuries. Forty were in the upper extremity ; sixty were in the lower extremity.

There were four amputations for gangrene, all in the lower extremity.

There was one amputation for cellular inflammation in the upper extremity.

There was one amputation for atrophy and deformity in the upper extremity of the right side.

There were eleven amputations for causes not stated. Four in the upper extremity, and seven in the lower extremity.

Of fifty-three amputations in the upper extremity, seventeen were of the right, twenty of the left, and sixteen not referred to either side.

Of one hundred and thirty-one amputations in the lower extremity, forty-nine were of the right, forty-two of the left, and forty not referred to either side.

Of the forty-one amputations whose result was fatal, ten were in the upper extremity ; thirty-one were in the lower extremity.

DEATHS.

Of the thirty-nine deaths which occurred, nine had for their immediate cause shock or collapse ; four on the first and five on the second day. Nine deaths were put down as due to exhaustion; five within the first five days, three between the fifth and the tenth days—one being in consequence of a double re-amputation of the legs on an old man 75 years of age—and one on the fourteenth day. Seven deaths were caused by septicæmia, one in consequence of a double amputation of the legs on the fourth day, the others on the sixth, eleventh, fifteenth, eighteenth, twenty-first and seventy-first days respectively. Fourteen were due to pyaemia, occurring respectively on the third, seventh, ninth, tenth, fourteenth, fifteenth, twenty-first, twenty-fifth, thirtieth, thirty-first, thirty-fifth, fifty-ninth and sixty-fourth days.

